# Extremely rare pediatric primary scrotum tumor: spermatic cord hemolymphangioma for a case report and literature review

**DOI:** 10.1186/s12957-023-03118-2

**Published:** 2023-07-26

**Authors:** Liang Liu, Yu Xiao, Xiao Yue, Qiang Wang

**Affiliations:** 1 Department of Urology, Baoding No. 1 Central Hospital, Changcheng North Street and Number 320, Bao-ding, 071000 Hebei China; 2 Prostate and Andrology Key Laboratory of Baoding, Changcheng North Street and Number 320, Bao-ding, 071000 Hebei China; 3 Psychosomatic Medical Center, The Fourth People’s Hospital of Chengdu, Chengdu, 610036 Sichuan China; 4 Psychosomatic Medical Center, The Clinical Hospital of Chengdu Brain Science Institute, MOE Key Lab for Neuroinformation, University of Electronic Science and Technology of China, Chengdu, 610036 Sichuan China

**Keywords:** Scrotum tumor, Scrotum surgery, Spermatic cord hemolymphangioma

## Abstract

Hemolymphangioma is an uncommon benign tumor type that commonly occurs in the head and neck. Primary spermatic cord hemolymphangioma (SCH) with only several reported, however, is extremely rare. Clinical diagnosis can be challenging because of its rarity. Although spermatic cord hemolymphangiomas are benign tumors, there is still a high recurrence rate in postoperative. A 15-year-old boy presented to our hospital with complaints of scrotal for 15 days and did not have other associated symptoms. The male genital color Doppler ultrasound revealed that a cystic echo in the left spermatic cord region and above the testes was about 32 mm × 20 mm × 14 mm. He underwent left en bloc scrotum tumor resection under general anesthesia, and pathologic examination showed SCH. He was discharged from the hospital in the second postoperative day. After 1-month follow-up, the patient recovered well without recurrence. The patient is currently in follow-up phase. Up to date, only a few cases have been reported in the literature about SCH. So, we hope to raise the awareness of the diagnosis of SCH in clinical practice although this case.

## Introduction

Hemolymphangioma, also known as angioma lymphaticum, is a congenital benign tumor of malformed blood and lymphatic vessels, not a true neoplasm that shows a mixture of blood vessels and lymphatics [1]. It is categorized into congenital and secondary tumors. The secondary tumors may be due to disorders of lymphatic reflux and damage to the lymph vessels during surgery or trauma, while the primary tumors were an occluded pathway resulting from abnormal development of the lymphatic during embryogenesis [2].

Hemolymphangioma occurs mostly in children, and its incidence rate was 1.2–2.8 per 1000 newborn infants [3]. Hemolymphangioma has been previously published in the oral region [4], axilla [5], extremities [6–8], orbit [9, 10], tongue [11, 12], esophagus [13], hepatica [14], stomach [15, 16], abdomen [17], rectum [18], pericardium [19], duodenum [20], small intestine [21], and spleen [21–25] and adrenal gland [26]. However, primary hemolymphangioma originating in the spermatic cord tumor is extremely rare. To the best of our knowledge, this is the second primary spermatic cord hemolymphangioma (SCH) case reported in the literature according to PubMed database (http://www.ncbi.nlm.nih.gov/pubmed; accessed on 13 May 2023).

SCH is extremely rare, which could readily misdiagnose with other diseases such as spermatic cord tuberculosis, spermatic cord cyst, and spermatic cord hydrocele. There are no standard treatments for SCH. SCH is rarely a benign disorder, which may invade the surrounding organs, and it has a high rate of local recurrence.

Therefore, in this paper, we describe the first complete en bloc surgical tumor resection for SCH and show favorable results. In this case report, we aim to increase awareness of this rare condition and share our experience with diagnosis.

## Case presentation

### Clinical diagnosis of SCH

**Fig. 1:**
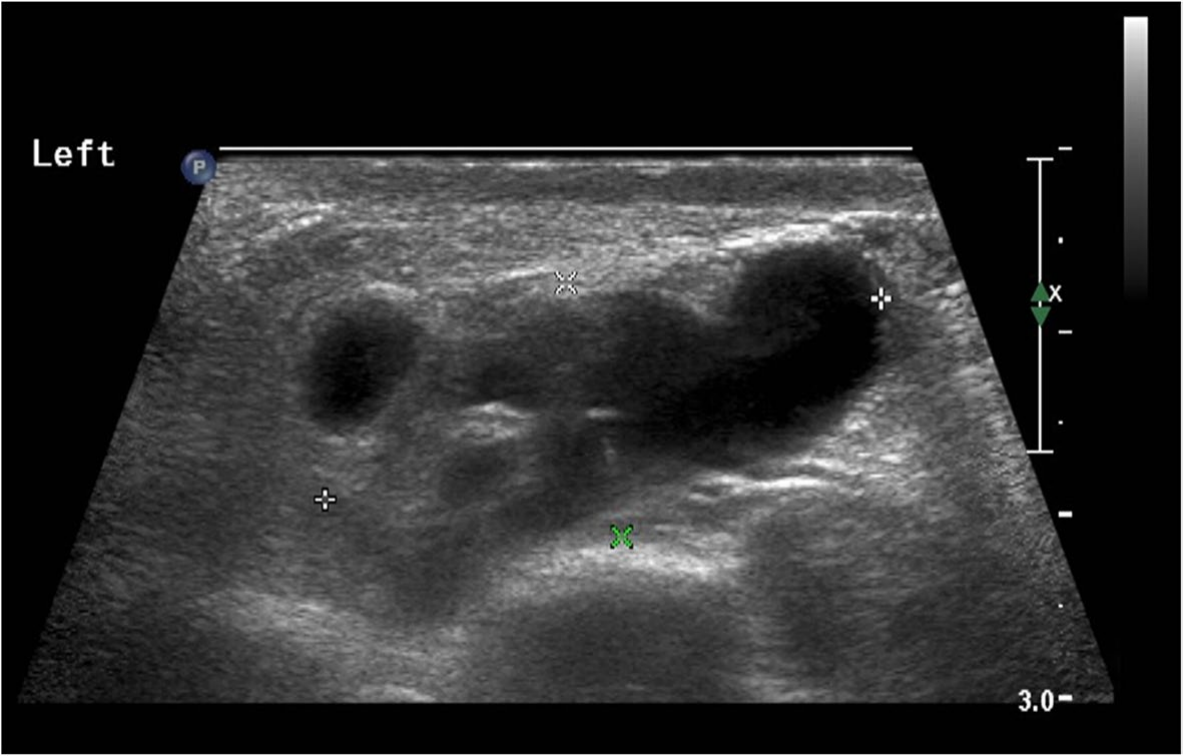
A cystic echo in the left spermatic cord region and above the testes is about 32 mm × 20 mm × 14 mm

A 15-year-old boy presented to our hospital with complaints of scrotal for 15 days and did not have other associated symptoms on 28 March 2023. The male genital color Doppler ultrasound revealed that the root of the scrotum on the left side and inguinal area was anechoic before 4 days admission in other hospital. Physical examination is as follows: T 36.0 °C. There was a round mass about 25 mm × 20 mm, with a smooth surface and good elasticity, arising from the root of the scrotum on the left side. Alpha‐fetoprotein (AFP): 1.13 ng/mL, carcinoembryonic antigen (CEA): 3.15 ng/mL, human chorionic gonadotropin (β-hCG): 0.10 mIU/ml, lactate dehydrogenase (LDH): 142.80 U/L, and erythrocyte sedimentation rate (ESR): 3 mm/h. Tuberculoid-specific cellular immune responses and tuberculosis antibody test were negative. The male genital color Doppler ultrasound revealed that a cystic echo in the left spermatic cord region and above the testes was about 32 mm × 20 mm × 14 mm (Fig. 1). The left scrotum tumor diagnosis was before surgery.

### Pathologic diagnosis and treatment of SCH

**Fig. 2:**
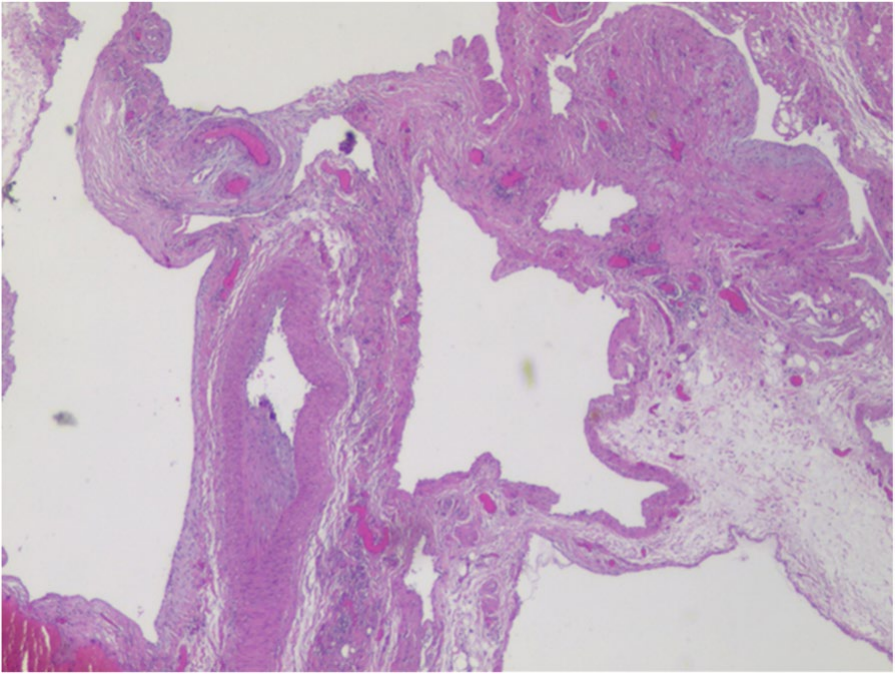
Most luminal tissues were dilated in the tumor tissue submitted

The patient underwent left en bloc scrotum tumor resection with preservation of spermatic vessels under general anesthesia. During the operation, the tumor, located within the spermatic cord region, appeared as a cystic mass (measured 35 mm × 20 mm × 20 mm) that was elastic, soft, and homogeneous with a light black area, with an irregular shape and irregular margins. Postoperative patient underwent anti-infection, detumescence, and other drug treatments. In order to define the tumor was removed completely, scrotal ultrasonography was performed again, and no obvious abnormalities were observed. The pathological result indicated that most luminal tissues were dilated in the tumor tissue submitted (Fig. 2). Immunohistochemical is as follows: Ki-67 (< 1%), CD34 (+), CD31 (+), and D2-40 (−). The final pathologic examination showed spermatic cord hemolymphangioma. Postoperative diagnosis was primary left spermatic cord hemolymphangioma.

### Followed-up and prognosis of SCH

The patient was discharged from the hospital in the second postoperative day, with no complications. After 1-month follow-up, the patient recovered well without recurrence. Due to the high recurrence rate, 6-month assessment is advised after post-operative and then annually thereafter until 3 years after the operation. Shortening the interval of follow-up time should be emphasized after partial surgical resection of the tumor. The patient is currently in follow-up phase.

## Discussion

Our case seems to be the first reported case of complete en bloc surgical tumor resection for SCH to the best of our knowledge.

Hemolymphangioma is a rarely benign neoplasm arising from the mesenchymal tissue, which could most commonly arise in the oral and maxillofacial head and neck regions [27]. However, spermatic cord hemolymphangioma is extremely rare. Only one patient of SCH was reported from 1960 to 2023 in the literature [28]. Tumors of the hemolymphangioma can be separated into primary and secondary forms. Our patient denied any history of trauma or surgery, so we wanted to highlight the tumor that should be considered to originate in the spermatic cord tissues and classified as a primary tumor.

Using PubMed, we searched by “hemangiolymphangioma [All Fields]” OR “hemolymphangioma [All Fields]” and “Spermatic Cord [MH]” OR “Cord, Spermatic [All Fields]” OR “Cords, Spermatic [All Fields]” OR “Spermatic Cords [All Fields]” OR “Funiculus Spermaticus [All Fields]” the literature published through May 2023. Only one case report of SCH was found [28]. The patient, 17-year, had a tumor in the right inguinoscrotal region, and he was successfully treated with simultaneous orchiectomy. The postoperative pathology confirmed a diagnosis of hemolymphangioma, and he was diagnosed with recurrent SCH.

The onset of SCH can be insidious and requires high clinical suspicion. Color Doppler ultrasound, CT, and MRI are useful imaging modalities in the diagnosis of this disease. The color Doppler ultrasound can be effectively found in a cystic echo pattern. Cysts have an irregular shape, smooth surface, and unclear boundaries within the tubular anechoic region. The CT scan and MRI are both universal imaging techniques used to determine tumor extent and the invasion of the tumor. Hemolymphangiomas are usually cystic solid or solid tumors. Solid tissue may represent remnants and compressed vascular tissue, while cystic tissue results from ruptured and fused lymphatic vessels, of which depend on the composition of the blood vessels and whether co-infection or bleeding [23]. The use of MRI can aid in determining the association between the hemolymphangioma and the surrounding tissues and the degree to which it has invaded. The tumor could manifest as heterogeneous isointense on T1WI and hyper‑intensity on T2WI [29]. MRI and CT scans can contribute to the selection of surgical strategy and follow-up treatment. The definitive diagnosis, however, should be determined by histological examination.

In spite of hemolymphangioma’s benign nature, recurrence and invasion of adjacent organs have been reported [30]. To date, surgical resection of tumors of the hemolymphangioma has been the best therapeutic method. Operation types are determined by factors such as the size and location of the tumor and the proximity of the surrounding organs. If possible, the tumor should be completely removed whenever possible without damaging the surrounding tissue. A follow-up is necessary after surgery to determine whether the tumor has recurred or metastasized [31]. The recurrence rate has been reported to be 50–100% following a partly resected tumor, while complete surgical resection was only 10–27% [32]. In this case, although the tumor has an irregular outline, complete surgical resection of the tumor was performed successfully. There has been no recurrence in the follow-up so far. The diagnosis of SCH was reliably based on preoperative examination, and the mode of surgery should be as determined as possible preoperative. Imaging with color Doppler ultrasonography and CT is useful in clinics for determining the tumor’s size, location, properties, and the relation of tumor growth to the surrounding tissue before surgery, which could provide a more reliable basis for surgery.

## Conclusions

SCH is an extremely rare benign tumor. The spermatic vein was kept intact, and the tumor was completely resected in our case. Hence, as of now, there are almost no cases of complete excision of the tumor in patients with SCH. In our patient, provide new insights into SCH prognosis and complete excision of the tumor for SCH cases.

## Data Availability

The data presented in this study are available on request from the corresponding author. The data are not publicly available due to consideration for the patients’ privacy.
